# The Build-up of Auditory Stream Segregation: A Different Perspective

**DOI:** 10.3389/fpsyg.2012.00461

**Published:** 2012-10-31

**Authors:** Susann Deike, Peter Heil, Martin Böckmann-Barthel, André Brechmann

**Affiliations:** ^1^Special Lab Non-invasive Brain Imaging, Leibniz Institute for NeurobiologyMagdeburg, Germany; ^2^Department of Auditory Learning and Speech, Leibniz Institute for NeurobiologyMagdeburg, Germany; ^3^Department of Experimental Audiology, Otto-von-Guericke-University MagdeburgMagdeburg, Germany

**Keywords:** auditory streaming, perception, build-up, psychoacoustics, default one-stream percept

## Abstract

The build-up of auditory stream segregation refers to the notion that sequences of alternating A and B sounds initially tend to be heard as a single stream, but with time appear to split into separate streams. The central assumption in the analysis of this phenomenon is that streaming sequences are perceived as one stream at the beginning by default. In the present study, we test the validity of this assumption and document its impact on the apparent build-up phenomenon. Human listeners were presented with ABAB sequences, where A and B were harmonic tone complexes of seven different fundamental frequency separations (Δ*f*) ranging from 2 to 14 semitones. Subjects had to indicate, as promptly as possible, their initial percept of the sequences, as either “one stream” or “two streams,” and any changes thereof during the sequences. We found that subjects did not generally indicate a one-stream percept at the beginning of streaming sequences. Instead, the first perceptual decision depended on Δ*f*, with the probability of a one-stream percept decreasing, and that of a two-stream percept increasing, with increasing Δ*f*. Furthermore, subjects required some time to make and report a decision on their perceptual organization. Taking this time into account, the resulting time courses of two-stream probabilities differ markedly from those suggested by the conventional analysis. A build-up-like increase in two-stream probability was found only for the Δ*f* of six semitones. At the other Δ*f* conditions no or only minor increases in two-stream probability occurred. These results shed new light on the build-up of stream segregation and its possible neural correlates.

## Introduction

In recent years, the perceptual phenomenon of auditory stream segregation, i.e., the decomposition of a mixture of sounds into meaningful objects or streams, has been extensively investigated. In laboratory experiments, the mixture generally consists of A and B sounds, which differ in some specific characteristic(s), most often in frequency. Most studies used one of the two canonical streaming paradigms, which involve the consecutive presentation of A and B sounds, either in alternation (ABAB), a paradigm introduced by Miller and Heise (1950), or in triplets (ABA_), introduced by van Noorden (1975). Depending on the specific stimulation parameters, i.e., the physical differences between A and B sounds (for example, the difference in frequency) and their temporal separation, three different perceptual domains can be distinguished by means of the dominant perceptual organization. With small physical differences, A and B sounds are predominantly heard as a single stream, whereas large physical differences and high tone presentation rates lead to the percept of two segregated streams. With intermediate stimulus parameters, both of these percepts are possible (ambiguous domain) and the listener can switch between them.

Current research on auditory stream segregation focuses on the stimulus characteristics sufficient for stream segregation to occur (for review, see Moore and Gockel, 2002), on the underlying neural mechanisms in different species including humans (for recent reviews, see Carlyon, 2004; Micheyl et al., 2007; Snyder and Alain, 2007; Bee and Micheyl, 2008; Shamma and Micheyl, 2010), on the development of conceptual and computational models accounting for psychophysical and physiological data (Hartmann and Johnson, 1991; Beauvois and Meddis, 1996; Denham and Winkler, 2006; Winkler et al., 2009; Shamma et al., 2011), and on the temporal dynamics of auditory stream segregation. The latter includes the build-up of stream segregation (Bregman, 1978; Anstis and Saida, 1985; Cusack et al., 2004; Micheyl et al., 2005; Pressnitzer et al., 2008; Bee et al., 2010; Haywood and Roberts, 2010) and the percept stability (Pressnitzer et al., 2008; Bendixen et al., 2010; Kondo et al., 2012; Denham et al., in press). The present study focuses on the build-up of stream segregation.

The build-up of stream segregation refers to the notion that sequences of sounds are thought to be initially heard as a single stream, and that with time the same sounds appear to split into two separate streams which the listener can follow individually (see, e.g., Anstis and Saida, 1985; Micheyl et al., 2005). The key assumption made in all studies concerned with the build-up, namely that all sound sequences are initially heard as a single coherent stream, drives the data analyses as well as their interpretation. For example, Bregman (1978) argued from a cognitive perspective that a certain amount of evidence must have been accumulated over several seconds by the auditory system, before it interprets the input as originating from two sound sources rather than one. Anstis and Saida (1985) suggested the build-up to be based on the adaptation of frequency-change detectors that results in a long-term trend over time toward a two-stream percept. Micheyl et al. (2005) also suggested that a longer-term decay in neural responses (referred to as “habituation”) is the key mechanism underlying the build-up. They proposed that the build-up of stream segregation arises “simply as a by-product of sound-event detection within frequency-specific, but otherwise unspecialized, neural populations in A1 and their habituation.”

Notably, however, the assumption of a default one-stream percept at the beginning of the sound sequences, prevalent in all of these hypotheses and in data analyses, has never been directly tested. Therefore, the present study aims at providing a test for this assumption by directly measuring the emergence of both the one-stream as well as the two-stream percept. Our results shed new light onto the build-up phenomenon.

## Materials and Methods

### Subjects

Twenty-two listeners (9 male and 13 female), aged between 19 and 38 years, participated in the experiments. All subjects had normal audiograms, with absolute thresholds ≤20 dB hearing level. The subjects gave written informed consent to the study which was approved by the Ethics Committee of the Otto-von-Guericke University of Magdeburg.

### Apparatus, stimuli, and procedure

The psychophysical measurements were performed in an acoustically shielded chamber (Industrial Acoustic Chambers, Niederkrüchten, Germany). The stimuli, which were digitally synthesized in Matlab (The Mathworks Inc., Natick, MA, USA), were harmonic tone complexes comprising the fundamental frequency, *F*_0_, and four partials with frequencies from 2 to 5 *F*_0_. All partials started and ended simultaneously and had equal amplitude. Each tone complex lasted 25 ms including 3.8 ms cosine-squared onset and offset ramps. The tone complexes were presented in ABAB sequences of 30-s duration with a presentation rate of 6 Hz. This rate was chosen based on the results of a former study on stream segregation by Deike et al. (2010) in which similar stimuli were used. In general, presentation rates in the range from 1 to 10 Hz have been commonly used in streaming experiments (e.g., Sussman et al., 1999; Vliegen and Oxenham, 1999; Cusack, 2005; Gutschalk et al., 2005; Micheyl et al., 2005; Snyder et al., 2006). A and B tone complexes differed in *F*_0_. In different conditions, seven frequency separations (Δ*f*) between the *F*_0_ of A and B tone complexes were used, viz., 2, 4, 6, 8, 10, 12, and 14 semitones. These Δ*f* values were achieved by varying the *F*_0_ of both the A and B tone complexes between conditions and relative to a *F*_0_ of 392 Hz. In this way, the subjects were prevented from getting familiar with a specific frequency, which might have biased their percept toward the two-stream one. In addition, within each condition, individual exemplars of both A and B tone complexes varied in *F*_0_, differing from the geometric mean by 0, ±1, and ±2 semitones (*F*_0_ variants). Table 1 lists all the *F*_0_ values used and their organization into A and B tone complexes. Within sequences, the different *F*_0_ variants were presented randomly and with equal probability. The assigned Δ*f* values therefore represent (geometric) mean *F*_0_ separations between A and B tone complexes. For each of the seven Δ*f* conditions, five different random sequences of A and B tone complexes were presented twice each, resulting in the presentation of 10 sequences per Δ*f* condition during the experiment. The different sequences were presented in pseudo-random order and alternated with silence of 10-s duration. The stimuli were presented binaurally via headphones (Sennheiser, HD 465) at an individually adjusted, comfortable sound level, using Presentation (Neurobehavioral Systems Inc., San Francisco, USA).

**Table 1:** **Fundamental frequencies (*F*_0_) of the tone complexes and their assignment to A and B sequences**.

	*F*_0_ variants from geometric mean (in semitone differences)					Condition (Δ*f* in semitones)
	−2	−1	0	+1	+2	
A tones (in Hz)	523	554	587	622	659	14
	494	523	554	587	622	12
	466	494	523	554	587	10
	440	466	494	523	554	8
	415	440	466	494	523	6
	392	415	440	466	494	4
	370	392	415	440	466	2
B tones (in Hz)	330	349	370	392	415	2
	311	330	349	370	392	4
	294	311	330	349	370	6
	277	294	311	330	349	8
	262	277	294	311	330	10
	247	262	277	294	311	12
	233	247	262	277	294	14

Prior to the psychophysical measurements, the subjects received written instructions and additional verbal explanations if necessary. The subjects were asked to listen to the sound sequences and to continuously indicate their current percept by pressing the left mouse button with their right index finger when they perceived the low- and high-*F*_0_ tone complexes as one coherent stream, and the right mouse button with their right middle finger when they perceived them as two separate streams, i.e., when they heard a low and a high stream in parallel. The subjects were encouraged to indicate as promptly as possible after the onset of each sequence whether they heard one stream or two streams and to update their response every time the percept switched, until the end of the sequence. The type of all button presses and their timing relative to sequence onset were recorded. All subjects performed the experiment twice on two different days to assess test-retest reliability. To familiarize the subjects with the sound sequences and the task, they were exposed to two sequences prior to the actual measurements. The two familiarizing sequences employed the 2 and the 14 semitone Δ*f* conditions, which are most likely to promote one or the other perceptual alternative, i.e., the one-stream and the two-stream percept, respectively.

### Data analysis

As mentioned in the introduction, the common analysis of the build-up of stream segregation is based upon the assumption of a default one-stream percept at the beginning of streaming sequences. For the purpose of comparison, the initial analysis was conducted according to the literature (Cusack et al., 2004; Micheyl et al., 2005; Pressnitzer et al., 2008). In addition, however, the data were analyzed without making the assumption of a default one-stream percept. For better readability, a detailed description of the above mentioned analyses is provided in the result section.

## Results

### Test-retest reliability

**Figure 1:**
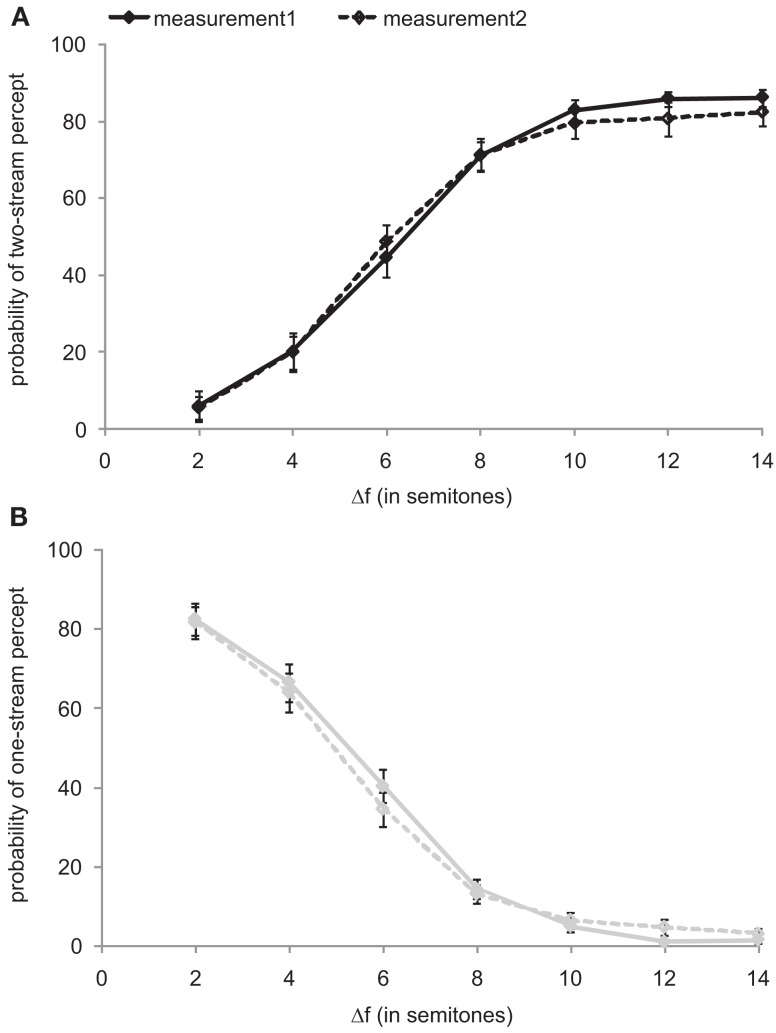
**Test-retest reliability**. The psychometric functions from the first (solid line, filled symbols) and the second measurement (dashed line, open symbols) represent the proportions of time that the stimulus sequences were perceived as two streams **(A)** and as one stream **(B)**, as functions of the frequency separation, Δ*f*, between **A** and **B** sounds (expressed as semitones). Symbols and error bars represent means and SEM. The proportions depend on Δ*f*, but do not differ between the two measurements.

Each subject performed the experiment twice, on different days. This allowed exploration of the test-retest reliability. For this purpose, we calculated for each subject and Δ*f* condition and for both measurements the proportions of time that the sound sequence was perceived as one stream and as two streams. Figure 1 plots these proportions, averaged across the 10 sequence presentations at each Δ*f* and across all 22 listeners, as a function of Δ*f*. In both measurements, the proportion of a two-stream percept increases with increasing Δ*f* (Figure 1A) and that of a one-stream percept decreases with increasing Δ*f* (Figure 1B). The comparison of these proportions between the two measurements revealed no significant differences in any Δ*f* condition (Wilcoxon signed-rank tests: all *p* > 0.1 for the one-stream percept and *p* > 0.3 for the two-stream percept). Because of this high test-retest reliability, the data of both measurements were pooled for further analyses.

### First percept

**Figure 2:**
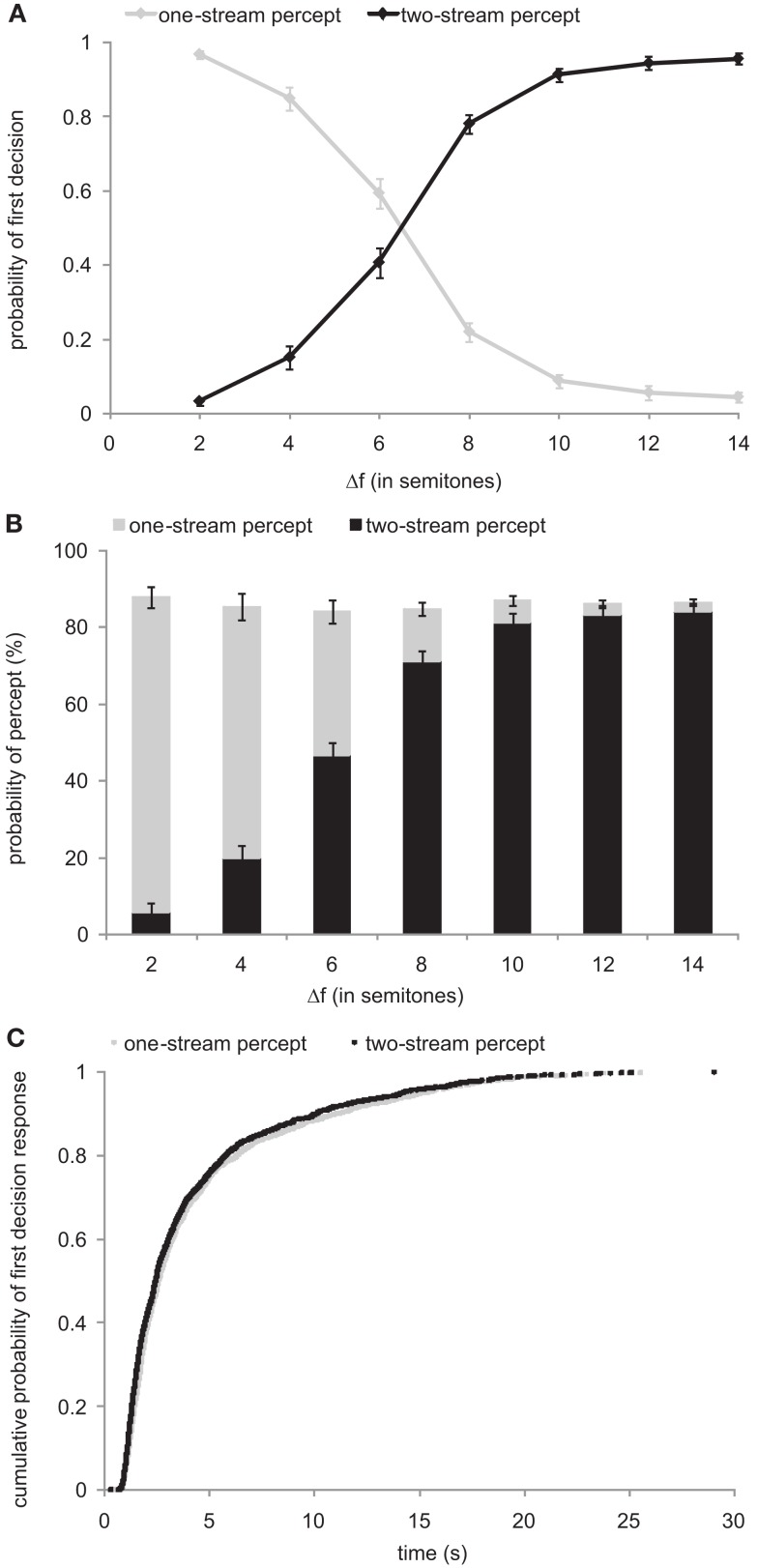
**Absence of a default one-stream percept**. The functions in **(A)** show the probability that the first decision is that of a one-stream percept (gray line) or that of a two-stream percept (black line), plotted as a function of Δ*f*. Symbols and error bars represent mean and SEM. **(B)** Proportions of time that the stimulus sequence was perceived as two streams (black bars) and as one stream (gray bars). Data were pooled over the first and second measurement. Note that the summed proportions do not add up to 1. This “missing” proportion represents the time from stimulus onset until the first perceptual decision was reported. **(C)** Cumulative distributions of latencies to the first perceptual decision, separated according to the decision (one-stream percept, *N* = 1234 or two-stream percept, *N* = 1833). The distributions were derived by combining latencies across all subjects and measurements.

We encouraged our subjects to indicate as promptly as possible after the onset of each sequence whether they perceived the tones as belonging to one stream or to two streams. Under the default assumption of a one-stream percept at the beginning, subjects should have always, or nearly always, indicated a one-stream percept first (by pressing the left mouse button). However, this was not the case. Figure 2A plots the probabilities that the first decision was in favor of a one-stream percept (gray line) or a two-stream percept (black line) as a function of Δ*f*. The panel shows that the probability that this decision was in favor of “one stream” is high for small Δ*f*, but declines to values near 0 for large Δ*f*. Conversely, the probability that this decision was in favor of two streams is low for small Δ*f* and increases to values near 1 for large Δ*f*. The correlation between the initial decision and Δ*f* is highly significant (Spearman’s correlation: |ρ| = 1; *p* < 10^−5^).

Furthermore, when we added up the proportions of time (out of the 30 s of each sequence) that the subjects indicated perceiving the tone sequences as one stream or as two streams, the sums were less than 1 (about 0.85–0.9), at all Δ*f* (Figure 2B). This means that during the “missing” proportion of time the stimulus sequence was neither reported to be perceived as one stream nor as two streams. In our experiment, this “missing” proportion corresponds to the initial time after sequence onset, where no decision on the perceptual organization was made.

In addition, we analyzed whether this initial time taken to make a first perceptual decision differed between the two decisions. Under the assumption of a default one-stream percept one would expect shorter latencies for the one-stream percept than for the two-stream percept. However, this was not the case. Figure 2C plots the cumulative distributions of the latencies, combined across subjects and measurements, for reporting an initial one-stream and a two-stream percept. The two distributions are very similar.

### Consequences for build-up

The data described above do not support the assumption of a default one-stream percept at the beginning of sound sequences. Taking this into account may have significant implications for the build-up phenomenon. This is examined here.

**Figure 3:**
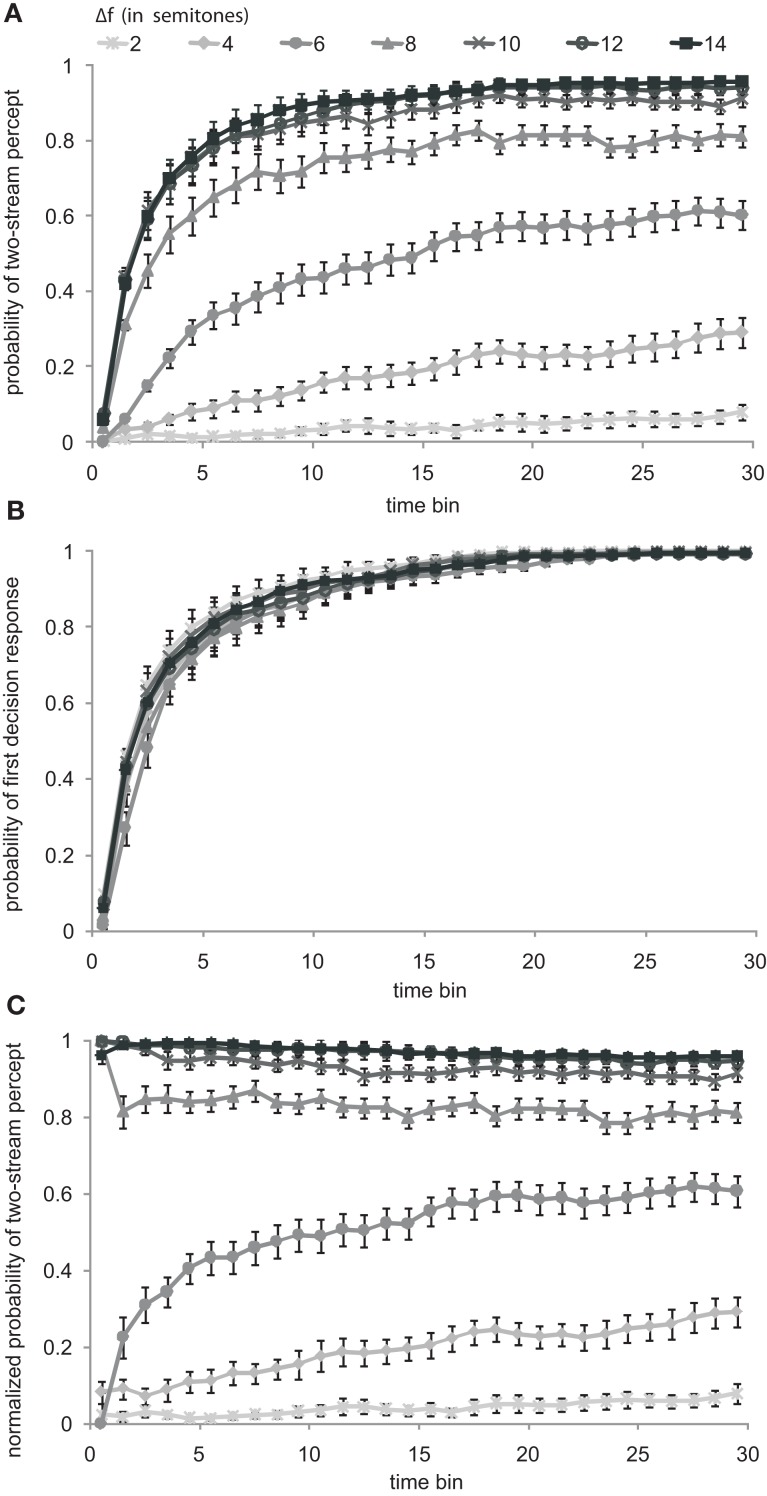
**Build-up of stream segregation: conventional versus normalized analysis**. **(A)** Probabilities of the two-stream percept are shown as functions of time (bin size 1 s) from sequence onset, for all tested Δ*f* conditions (gray scaled). The probability values were plotted at the center of the 1-s bins. Symbols and error bars represent mean and SEM. All functions show an asymptotic increase that has been interpreted as reflecting a build-up of stream segregation. The apparent build-up is strongest for the large semitone intervals. **(B)** Probabilities of the occurrence of the first response, representing the initial perceptual decision, are shown as functions of time from sequence onset. **(C)** Probabilities of the two-stream percept as in **(A)** but normalized by the probability that a first response has been made at all, as shown in **(B)**. Conventions in **(A)** also apply to **(B,C)**.

To allow comparison with existing data, we first calculated the probability of a two-stream percept in the conventional way (Cusack et al., 2004, Figure 3; Micheyl et al., 2005, Figure 1B; Pressnitzer et al., 2008, Figure 3). Each 30-s sequence was divided into 1-s bins. The value assigned to each bin was either 0 or 1, 0 for a left button press indicating a one-stream percept and 1 for a right button press indicating a two-stream percept. Subsequent values remained unchanged until the next button press occurred. The initial bins, before the first button press, were assigned a value of 0, i.e., assuming a default one-stream percept. From each of the ten presentations of the same sequence in corresponding bins we determined the probability of a two-stream percept across subjects and sessions. Figure 3A shows the grand average and the SEM resulting from this “conventional analysis.” An apparent build-up of a two-stream percept is observed. With increasing Δ*f*, the probability of a two-stream percept increases more rapidly with time and reaches higher maximum values. Both observations agree with previous studies, e.g., by Micheyl et al. (2005, Figure 1B), Pressnitzer et al. (2008, Figure 3), and Cusack et al. (2004, Figure 3).

This conventional analysis, however, does not take into account the fact that the subjects needed some (variable) time to make a decision regarding their first percept. Instead, the conventional analysis makes the tacit assumption of a default one-stream percept right from the start of the sequence. Even if a subject’s first decision would have always been that of a one-stream percept (unlike what we find here), it is necessary to correct for the time required to make, and report, such a decision. This is accomplished by normalizing (i.e., dividing) the probability of a two-stream percept (shown in Figure 3A) by the probability that a first perceptual decision has been made at all (shown in Figure 3B, separately for each of the seven Δ*f* conditions). This probability increases in an exponential-like fashion, reaching 0.5 after 2–3 s and 1 near the end of the sequence, in a similar way in the different Δ*f* conditions.

Figure 3C presents the two-stream probability functions, resulting from this “normalized analysis.” These normalized functions are markedly different from the conventional build-up functions of Figure 3A. The latter imply a build-up in all Δ*f* conditions which is most rapid and most pronounced for the large Δ*f* values (8, 10, 12, and 14 semitones). In contrast, the normalized functions for the same large Δ*f* values suggest no build-up at all. If anything, the probability of a two-stream percept decreases slightly with time from sequence onset. In the remaining Δ*f* conditions (2, 4, and 6 semitones), a build-up appears to be present, but it is less pronounced than suggested by the conventional analysis.

In summary, with the normalized analysis, i.e., after accounting for the fact that subjects require some time to make, and report, a decision on their first percept of the stimulus sequences (as one stream or as two streams), the probability of a two-stream percept increases with time from sequence onset only in small and intermediate Δ*f* conditions (2–6 semitones). In large Δ*f* conditions (8–14 semitones), this probability does not increase, unlike what would be expected from a build-up. Instead, and in marked contrast to the outcome of the conventional analysis, the probability of a two-stream percept decreases with time from sequence onset.

## Discussion

The present study tested the assumption of a default one-stream percept at the beginning of streaming sequences prevalent in the conventional analysis and elucidated its consequences for the analysis of the build-up phenomenon. Our data do not provide an empirical basis to assume a default one-stream percept. Instead, the first perceptual decision depended on the frequency separation between A and B sounds. In addition, we did not find shorter response latencies for the one-stream percept than for the two-stream percept, unlike what would have been expected under the default assumption of a one-stream percept at the beginning of sound sequences. Consequently, it is necessary to analyze the emergence of the two-stream percept over time without making the default one-stream assumption.

### Does build-up exist?

The present study analyzed the build-up of stream segregation by considering the effectively measured probabilities of a two-stream percept at each time point. The resulting functions of the two-stream probability over time differ from those obtained by the conventional analysis. For large frequency separations (8, 10, 12, and 14 semitones), the normalized functions did not show a build-up of a two-stream percept over time, if anything, they suggest a decrease (see Figure 3C). This is in marked contrast to the functions obtained with the conventional analysis which suggest a rapid and pronounced increase of the probability of a two-stream percept at these frequency separations (see Figure 3A). Only for small and intermediate frequency separations (2, 4, and 6 semitones), an increase in the probability of a two-stream percept was observed with the normalized analysis (see Figure 3C). However, at the two smallest frequency separations, the probability of a two-stream percept increased only slightly and the one-stream percept dominated at all times. Therefore, it may be questioned whether this subtle increase does reflect a true build-up. It should be pointed out that currently there is no agreed-upon quantitative criterion to define a build-up.

At the frequency separation of six semitones, our results show the most pronounced change in perceptual organization from a one-stream dominance at the beginning to a balance of both percepts at the end of the sequence which indicates perceptual ambiguity. Such ambiguity may be crucial for build-up to occur. This should be clarified in future studies or by reanalyzing data of other studies according to the normalized analysis proposed here.

### Relation to the neural basis of stream segregation

One current hypothesis concerning the neural underpinnings of auditory stream segregation suggests that frequency selectivity of tonotopically organized neurons in primary auditory cortex fields in combination with physiological forward suppression leads to separate representations of A and B tones (Fishman et al., 2001, 2004; Kanwal et al., 2003; Bee and Klump, 2004, 2005). The general idea is that the more A and B tones are represented in separate neural populations the more likely is a two-stream percept. With respect to the build-up phenomenon of stream segregation this hypothesis would predict that the representation of A and B tones in tonotopic maps should change over time showing an increasing separation of the neural populations responding to the A and to the B tones. This hypothesis was tested by Bee et al. (2010), Micheyl et al. (2005), and Pressnitzer et al. (2008). These studies computed neurometric functions using a thresholding model that describes the strength of neural responses to A and B tones in the primary auditory cortex (A1) of awake rhesus monkeys (Micheyl et al., 2005), in the cochlear nucleus of anesthetized guinea pigs (Pressnitzer et al., 2008), and in field L2 (homolog of mammalian A1) of awake starlings (Bee et al., 2010). The model predicts a one-stream percept if both the A and the B tones evoke above-threshold responses in the same neurons. In contrast, a two-stream percept is predicted if neurons tuned to the A tones exhibit above-threshold activity during the presentation of A tones but below threshold activity during the presentation of B tones. It was shown that at the beginning of stimulus sequences spike rates evoked by tones at and away from the best frequency (BF) exceed the threshold, resulting in a one-stream percept according to the model. Thereafter, the responses start to decrease due to multi-second adaptation and the weaker spike rates evoked by tones away from the BF eventually fall below the threshold, while the higher spike rates evoked by BF tones remain above it, resulting in a two-stream percept according to the model. Micheyl et al. (2005) and Pressnitzer et al. (2008) adjusted the threshold such that these neurometric functions measured in monkeys and guinea pigs, respectively, fitted best the psychometric functions measured in human listeners. They showed that the time course and extent of neural multi-second adaptation is compatible with psychophysical measurements of the build-up of auditory stream segregation in human listeners. Both neurometric and psychometric time courses showed an influence of Δ*f* on the rate of increase of the probability of a two-stream percept over time. This rate of increase was highest at large Δ*f* values and dropped to zero at small Δ*f* values. However, at large Δ*f* values the neurometric functions attained higher probabilities than the psychometric functions at early time points from 1 to 3 s (see Micheyl et al., 2005, Figure 4; Pressnitzer et al., 2008, Figure 3). With the normalized analysis of the psychometric functions as proposed here, this discrepancy might be reduced, because at large Δ*f* values our approach leads to higher probabilities of the two-stream percept in this time range. Up to 1 s, however, essentially all decisions of our subjects were in favor of a two-stream percept which seems to be inconsistent with the neuronal responses at the start of the sequence where both tones exceed threshold at large Δ*f* values (Micheyl et al., 2005; Pressnitzer et al., 2008). To resolve this discrepancy it is important to consider the time subjects need to make the first decision. In our case, only 2 of 3080 possible responses across subjects, measurements, sequence presentations, and conditions were actually made within the first 500 ms. Thus, in contrast to the study by Micheyl et al. (2005) und Pressnitzer et al. (2008) our psychometric function is actually not defined at time point 500 ms which is due to the fact that we do not assume a default one-stream percept. We would argue that any link between psychometric and neurometric data is only reasonable at time points where both functions are defined.

## Conflict of Interest Statement

The authors declare that the research was conducted in the absence of any commercial or financial relationships that could be construed as a potential conflict of interest.
